# The Associations of Serum Folate Forms with Metabolic Dysfunction-Associated Fatty Liver Disease and Liver Fibrosis: A Nationwide Cross-Sectional Study

**DOI:** 10.3390/metabo15060370

**Published:** 2025-06-05

**Authors:** Hai Zhao, Wei Fan, Yan Yan, Yuxing Liu, Xuejun Kang

**Affiliations:** Key Laboratory of Child Development and Learning Science (Ministry of Education), School of Biological Science and Medical Engineering, Southeast University, Nanjing 211189, China; 230218247@seu.edu.cn (H.Z.);

**Keywords:** folate forms, hepatic fibrosis, hepatic steatosis, MAFLD, NHANES

## Abstract

**Background**: Accumulating evidence indicates a link between folate and metabolic dysfunction-associated fatty liver disease (MAFLD). **Objectives**: The aim of this study was to ascertain whether different serum folate forms are associated with newly defined MAFLD as well as liver fibrosis in the US general population. **Methods**: This cross-sectional study used data from the 2017–2020 (March) cycle and 2017–2018 cycle of the National Health and Nutrition Examination Survey (NHANES) in the US. Hepatic steatosis and fibrosis were evaluated by transient elastography, which employed controlled attenuation parameters and liver stiffness measurements as assessment indicators. **Results**: 7447 eligible individuals were included. The estimated prevalence of MAFLD and liver fibrosis was 51.6% (95% confidence interval [CI]: 50.4–52.7%) and 10.0% (95% CI: 9.3–10.7%). After adjusting for confounding factors, for every 1 nmol/L increase in serum 5-methyltetrahydrofolate (5-mTHF), the risk of developing liver fibrosis decreased by 1% (95% CI: 1–2%, *p* < 0.001), and the risk of developing MAFLD decreased by 1% (95% CI: 0–2%, *p* = 0.005). There were also significant differences in indicators such as alanine aminotransferase (ALT), gamma-glutamyl transaminase (GGT), and C-reactive protein (CRP) between the MAFLD group and the non-MAFLD group (all *p* values < 0.001). **Conclusions**: This study suggests the prevalence of MAFLD and liver fibrosis decreased significantly with the increase in serum 5-mTHF concentration.

## 1. Introduction

Metabolic dysfunction-associated fatty liver disease (MAFLD) is a chronic liver disease closely related to metabolic dysfunction, mainly characterized by hepatic fat accumulation. The introduction of MAFLD has brought about a notable transformation in liver disease classification by incorporating metabolic abnormality indicators, such as insulin resistance, heightened sensitivity to C-reactive protein (CRP), and other complex metabolic risk factors [1,2]. Metabolic dysfunction-associated steatotic liver disease (MASLD), historically termed non-alcoholic fatty liver disease (NAFLD), follows a complex and prolonged natural course. In a subset of patients, inflammation emerges alongside the risk of progressive fibrosis, which may ultimately lead to cirrhosis [3]. Traditional diagnoses like non-alcoholic steatohepatitis (NASH) and NAFLD rely on identifying hepatic steatosis via imaging or histological examinations, while excluding other causes of fatty liver disease, such as alcoholic liver disease, viral liver disease, and other drug-induced liver disorders. In contrast, MAFLD is no longer an exclusion-based disease but an inclusion-based one, with diagnostic criteria rooted in underlying metabolic abnormalities. This practical stance specifically excludes conditions like viral hepatitis, alcoholic liver disease, and other secondary liver disorders [4]. MAFLD attempts to identify those with fatty liver conditions by integrating metabolic markers. The individuals in question not only fulfill the traditional criteria for NAFLD but also show an increased likelihood of disease progression [5]. This advancement in the concept highlights a more in-depth understanding of the complex interaction between metabolic factors and liver health. It ushers in a more comprehensive and clinically relevant method for diagnosing and treating fatty liver disorders in the scientific community [6]. This new approach takes into account the complex relationship between metabolism and liver function, which enables a more accurate assessment of patients and potentially leads to more effective treatment strategies.

Folate serves as a general term that encompasses specific forms such as 5-methyltetrahydrofolate (5-mTHF), folic acid, and tetrahydrofolate (THF). Each of these forms has its own unique functions and roles [7]. 5-mTHF, a directly utilizable active form in the body, is vital in various cellular processes. It participates in various biological processes, such as gene regulation, neurotransmitter synthesis, and the detoxification process [8]. Folic acid needs to be transformed into active forms like 5-mTHF so that it can be utilized by the body. It is often taken by people as a supplement. It is an important component of women’s prenatal supplements, which can prevent neural tube defects in fetal development [9]. THF acts as an intermediate in the folate pathway. It is responsible for transporting one-carbon units, which are essential for the synthesis of nucleotides and amino acids. THF can be converted into different forms through enzymatic reactions [10]. In the research on NAFLD, it has been firmly established that folic acid is intricately involved in processes such as oxidative stress, lipid metabolism, and hepatitis [11]. Animal studies have revealed compelling evidence that folic acid supplementation can effectively alleviate hepatic steatosis [12,13]. Human studies have further corroborated the pivotal role of serum folic acid in preventing the progression of NASH. It has been discovered that folic acid possesses the remarkable ability to reduce liver fibrosis and mitigate inflammatory responses within the liver [14]. Additionally, epidemiological investigations have unearthed that serum folate and 5-mTHF serve as protective factors against NAFLD [15].

Previous studies exploring the relationship between different serum folate levels and NAFLD as well as liver fibrosis have employed a variety of analytical methods. Our main aim was to increase the sample size. To achieve this, we included participants who had undergone vibration-controlled transient elastography (VCTE) from two cycles of the National Health and Nutrition Examination Survey (NHANES): the 2017–2018 cycle and the 2017–2020 (March) cycle. Afterward, we embarked on an in-depth investigation of the cross-sectional associations. Specifically, we looked at the relationships between different serum folate levels and MAFLD as well as liver fibrosis.

## 2. Methods

### 2.1. Study Design

**Figure 1:**
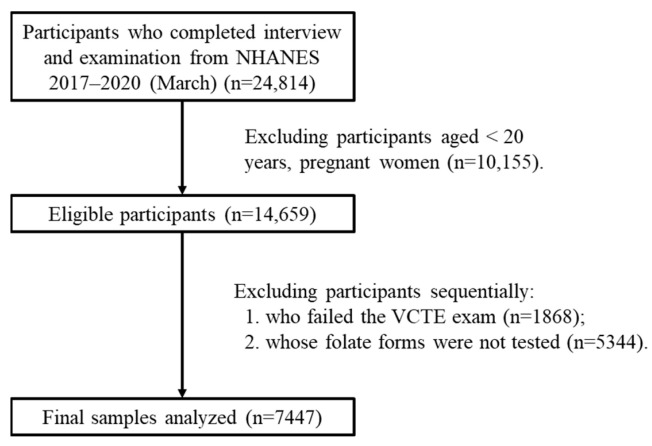
Flowchart of eligible participants included in this study. Abbreviations: VCTE: vibration-controlled transient elastography.

NHANES was approved by the research ethics review board of the US National Center for Health Statistics [16]. Since 1999, the survey, which includes questionnaires, physical assessments, and lab analyses, has been conducted biennially, and written informed consent has been obtained from each participant [17]. The data for this research were sourced from specific, independent subsets of NHANES, namely the released cycles spanning 2017–2018 and 2017–2020 (March). The study population was limited to adults aged 20 years and above. Initially, there were 24,814 participants. However, after applying the exclusion criteria, 7447 participants were finally included in the study (Figure 1). Self-reporting was used to collect information on demographic characteristics and lifestyle factors. The assessment of income was based on the ratio of family income to the poverty threshold, which was specific to the particular year, state, and family size [18]. In the Mobile Examination Center, height and weight were measured and utilized to compute the body mass index (BMI). Alcohol consumption was categorized into three types based on the past year’s consumption: never, sometimes (less than twice a week), and often (more than twice a week). Participants were informed by a doctor or health professional that they had hepatitis B or hepatitis C.

### 2.2. Diagnosis of MAFLD and Liver Fibrosis

Vibration-controlled transient elastography (VCTE), carried out using the FibroScan^®^ 502 Touch machine (Echosens, Paris, France), was employed to concurrently evaluate hepatic steatosis and fibrosis. This device provided two key outputs: the controlled attenuation parameter (CAP) and liver stiffness measurement (LSM), which served as markers for hepatic steatosis and fibrosis, respectively. In systematic reviews that compared VCTE with biopsy (the gold standard) for detecting severe liver fibrosis, the mean area under the receiver operating characteristic (ROC) curve was found to be 0.89 (95% confidence interval [CI]: 0.88–0.91). Additionally, the overall sensitivity and specificity were 82% (95% CI: 78–86%) and 86% (95% CI: 80–91%) [19,20]. Consistent with prior research, hepatic steatosis was identified when the median CAP was equal to or greater than 248 decibels per meter (dB/m). Clinically significant fibrosis was defined as a median LSM of at least 8.2 kilopascals (kPa) [21,22]. The newly introduced criteria for diagnosing MAFLD were grounded in the presence of hepatic steatosis along with at least one of the following: overweight/obesity, type 2 diabetes mellitus, or metabolic dysregulation [2].

### 2.3. Quantification of Serum Folate Forms and Oxidative Stress

The procedures regarding peripheral blood sampling, its storage, and the determination of folate forms were described meticulously [23]. Five folate forms, 5-methyltetrahydrofolate (5-mTHF), folic acid, tetrahydrofolate (THF), 5-formyltetrahydrofolate (5-fTHF), and 5,10-methenyltetrahydrofolate (5,10-mTHF), and an oxidation product of 5-methyltetrahydrofolate called MeFox (pyrazino-s-triazine derivative of 4-α-hydroxy-5-methyltetrahydrofolate) were measured by isotope-dilution high-performance liquid chromatography coupled with tandem mass spectrometry (LC-MS/MS) [24]. For analytes with results below the lower limit of detection (LLOD), the value was substituted with LLOD/√2 [25]. The LLODs for whole-blood trihalomethanes are shown in Table 1.

Serum C-reactive protein (CRP), a commonly used marker of inflammation, was measured by latex-enhanced nephelometry [26]. Serum alanine aminotransferase (ALT) and gamma-glutamyl transaminase (GGT) concentrations were also calculated.

### 2.4. Statistics

Means and standard deviations (SDs) were utilized to present continuous variables, while proportions were employed for categorical variables. The Chi-square test and Mann–Whitney U test were applied to compare the categorical and continuous variables, respectively. Logistic regression was conducted to discover the associations between serum folate forms and MAFLD as well as liver fibrosis. Three models were gradually conducted in order to adjust for possible confounding effects from various combinations of covariates. Model 1 was unadjusted, providing the crude odds ratio (OR) values. Model 2 was adjusted for age and gender. Model 3 was further adjusted for race/ethnicity, education level, family poverty–income ratio (PIR), BMI, waist circumference, and smoking status. All analyses were performed using IBM SPSS (version 22.0). A two-sided *p* value less than 0.05 was regarded as statistically significant.

## 3. Results

Table 2 presents the demographic characteristics of the 7447 participants, among whom the mean age was 48 years. Among those with MAFLD, 56.0% were female and 35.1% were white. In the hepatic fibrosis group, individuals with a college degree or higher accounted for 37.1% of the total. The proportion of MAFLD was higher in the wealthy (i.e., PIR > 3) compared to the poor (PIR ≤ 1). Smoking status also differed; 40.4% of those with hepatic fibrosis issues were current smokers. Other biochemistry indicators were statistically different between the MAFLD group and the non-MAFLD group (Table S1).

The overall prevalence of MAFLD and hepatic fibrosis in the general population aged 20 years and older was 51.6% (95% CI: 50.4–52.7%) and 10.0% (95% CI: 9.3–10.7%), respectively. MAFLD was more prevalent among females (37.3%, 95% CI: 36.1–38.6%) than males (31.9%, 95% CI: 30.6–33.2%). White people were the most susceptible race/ethnicity, with a prevalence of 27.2% (95% CI: 26.0–28.4%) for MAFLD. An increased susceptibility to MAFLD and hepatic fibrosis was observed in the study population and was linked to obesity and smoking status (Table 3).

We conducted logistic regression analysis to explore the associations between different folate forms and the risks of MAFLD and hepatic fibrosis (Table 4). In Model 1, without adjustment for confounding factors, for every 1 nmol/L increase in serum 5-mTHF, the risk of liver fibrosis decreased by 2% (95% CI: 1–2%, *p* < 0.001), and the risk of MAFLD decreased by 1% (95% CI: 1–2%, *p* < 0.001). This association remained after adjusting for confounding factors. For every 1 nmol/L increase in serum folic acid, the risk of liver fibrosis decreased by 11% (95% CI: 7–15%, *p* < 0.001) in Model 1 and even by 6% (95% CI: 0–11%, *p* = 0.043) in Model 3. THF levels also showed a significant association, which seemed to be a risk factor for hepatic fibrosis (OR = 2.41, 95% CI: 1.65–3.52, *p* < 0.001) and for MAFLD (OR = 1.76, 95% CI: 1.27–2.43, *p* = 0.001). The distribution of serum folate forms among study participants is shown in Table 5 and Table S2.

## 4. Discussion

Our research aimed to uncover the potential linkages between serum folate forms and MAFLD as well as hepatic fibrosis. We utilized a nationally representative sample of 7447 adults from the US. We discovered associations between the concentrations of serum 5-mTHF, folic acid, and THF and the likelihood of developing hepatic fibrosis. Moreover, a positive correlation was found between the concentration of serum 5-mTHF and the probability of developing MAFLD. Even after accounting for confounding covariates, these associations persisted and achieved statistical significance. The results suggested that serum 5-mTHF might have the potential to reduce the risk of developing MAFLD and hepatic fibrosis.

Our study found no significant relationship between the presence of folic acid and the prevalence of MAFLD, which is consistent with some previous studies on young adults [27]. However, certain contrasting studies have indicated a positive correlation between the concentration of serum folic acid and the incidence of NAFLD [15]. Elevated serum folic acid levels have been found to be associated with an increase in pro-inflammatory factors and a decrease in the cytotoxicity of natural killer cells. These changes might play a role in the development of NAFLD [28]. It is important to note that the definition of NAFLD differs across studies. This variance in defining NAFLD or MAFLD could explain the inconsistent results observed. In previous mouse studies, improvements in the intestinal microbiota were accompanied by a reduction in steatosis and an increase in hepatic THF content [29]. However, there has been a lack of in-depth experimental or epidemiological research to clarify the relationship between THF and hepatic steatosis. Our current study addresses this knowledge gap. We have uncovered a notable correlation between 5-mTHF, THF, and MAFLD. More research into the potential underlying mechanisms is crucial for a comprehensive understanding of this relationship.

The following studies may shed light on the potential mechanisms by which folate alleviates MAFLD. Firstly, folate is intricately involved in processes such as the synthesis of purines and adenosine. In the presence of folate deficiency, genes related to hepatic fat synthesis are upregulated. The excessive synthesis resulting from this upregulation may give rise to hepatic steatosis [30]. Secondly, a deficiency of folate can interfere with the fibroblast growth factor signaling pathway, which is closely associated with insulin resistance. The regulation in adipose accumulation by fibroblast growth factor contributes to the progression of lipotoxic liver disease [31,32]. Thirdly, folate can regulate the transcription of nicotinamide adenine dinucleotide phosphate oxidase [33]. The reduction in oxidative stress leads to an increase in adenosine monophosphate levels. Subsequently, liver kinase B1 is activated, which activates adenosine monophosphate-activated protein kinase in the liver [34]. These research results are consistent with our study. Our findings suggest that higher serum concentrations of 5-mTHF are associated with a lower prevalence of MAFLD.

Hepatic fibrosis is a pathophysiological process characterized by the abnormal proliferation of intrahepatic connective tissue caused by various pathogenic factors [35]. Any liver injury triggers a fibrotic process during hepatic repair and healing [36]. If the damaging factors cannot be removed long-term, the prolonged fibrotic process will progress to liver cirrhosis [37]. Therefore, it is not an independent disease but rather a consequence of the diffuse and excessive deposition of extracellular matrix (predominantly collagen) in the liver, leading to interstitial fibrosis. The etiologies of hepatic fibrosis are diverse, with viral hepatitis, alcoholic liver disease, fatty liver, and autoimmune diseases being common clinical causes [38]. In this study, alcohol consumption was not precisely quantified. Future research could focus on refining this aspect by incorporating detailed measurements of alcohol intake.

One of the merits of this study is that it delved into the relationship between six different forms of serum folate and MAFLD. Among them, 5-mTHF was identified as a protective factor, while THF was found to be a risk factor. We utilized a large, nationally representative sample from the US. This not only ensured the accurate assessment of the prevalence rates of MAFLD and liver fibrosis but also made the findings applicable to a diverse spectrum of American adults, regardless of their race or ethnic background.

## 5. Conclusions

Serum 5-mTHF was found to reduce the incidence of MAFLD and liver fibrosis. Conversely, THF emerged as a risk factor for both MAFLD and liver fibrosis. The concentration of serum folic acid was associated with a lower prevalence of liver fibrosis. Our research findings have been carefully adjusted to account for a wide range of potential confounding variables. This thorough adjustment further validates the reliability and strength of these associations. These results emphasize the critical importance of increasing public awareness about how elevated serum folate concentrations can influence metabolic health. It is essential to educate the public on this matter to promote better health outcomes and a deeper understanding of the role of folate in maintaining metabolic well-being.

## Figures and Tables

**Table 1:** The lower limit of detection for the six folate forms.

	LLOD (nmol/L)
serum 5-methyltetrahydrofolate (5-mTHF)	0.13
serum folic acid	0.14
serum 5-formyltetrahydrofolate (5-fTHF)	0.20
serum tetrahydrofolate (THF)	0.25
serum 5,10-methenyltetrahydrofolate (5,10-mTHF)	0.20
serum MeFox	0.10

Abbreviations: LLOD, lower limit of detection; MeFox, pyrazino-s-triazine derivative of 4-α-hydroxy-5-methyltetrahydrofolate.

**Table 2:** General characteristics of the study participants.

Characteristics	Total (*n* = 7447)	Hepatic Fibrosis (*n* = 746)	*p* ^a^	MAFLD (*n* = 3840)	*p* ^b^
age (year)	48 ± 17	55 ± 15	<0.001	51 ± 16	<0.001
20–39, %	36.5	18.9		27.9	
40–59, %	33.2	37.1		37.6	
≥60, %	30.3	44.0		34.5	
gender, female (%)	60.2	48.8	<0.001	56.0	<0.001
race/ethnicity (%)			0.004		<0.001
mexican american	13.1	14.6		16.3	
other hispanic	9.9	11.0		10.2	
non-hispanic white	33.9	36.9		35.1	
non-hispanic black	24.9	24.1		23.2	
other race	18.2	13.4		15.3	
education (%)			<0.001		<0.001
less than high school	17.8	20.9		18.2	
high school or equivalent	23.5	24.5		24.1	
college or above	33.5	37.1		34.9	
unknown	25.1	17.4		22.8	
PIR (%)			0.162		0.035
≤1	19.7	20.1		18.7	
1–3	42.4	45.3		43.8	
>3	37.9	34.6		37.5	
BMI (%)			<0.001		<0.001
underweight	5.0	2.6		n.a.	
normal weight	22.3	8.5		1.7	
overweight	30.9	17.2		34.2	
obese	41.8	71.8		64.1	
waist circumference (cm)	99.82 ± 17.34	115.83 ± 19.81	<0.001	109.83 ± 14.62	<0.001
smoking (%)			0.078		<0.001
never	55.2	59.6		61.9	
current	44.8	40.4		38.1	
drinking (%)			<0.001		<0.001
never	19.5	26.1		21.5	
sometimes	59.6	56.6		58.4	
often	21.0	17.3		20.1	
hepatitis B (%)	0.9	1.7	<0.001	1.1	0.074
hepatitis C (%)	1.6	5.0	<0.001	1.7	0.742
diabetes mellitus (%)	15.2	37.0	<0.001	24.3	<0.001
ALT (U/L)	21.21 ± 15.59	30.70 ± 25.74	<0.001	24.36 ± 17.67	<0.001
GGT (IU/L)	29.63 ± 45.72	56.53 ± 110.63	<0.001	34.34 ± 39.37	<0.001
CRP (mg/L)	4.11 ± 8.04	5.74 ± 8.68	<0.001	5.18 ± 9.13	<0.001
CAP (dB/m)	261.60 ± 62.16	307.43 ± 61.85	<0.001	307.47 ± 40.52	<0.001
LSM (kPa)	5.86 ± 5.01	14.83 ± 12.14	<0.001	6.58 ± 5.48	<0.001

Note: Data are presented as mean ± standard deviation (SD) for continuous variables and proportion for categorical variables. *p* values are calculated using the Chi-square test for categorical variables and the Mann–Whitney U test for continuous variables. *p* ^a^: The liver fibrosis group vs. the non-fibrosis group. *p* ^b^: The MAFLD group vs. the non-MAFLD group. Abbreviations: MAFLD, metabolic dysfunction-associated fatty liver disease; PIR, ratio of family income to poverty; BMI, body mass index; ALT, alanine aminotransferase; GGT, gamma-glutamyl transaminase; CRP, C-reactive protein; CAP, controlled attenuation parameter; LSM, liver stiffness measurement; n.a., not applicable.

**Table 3:** Prevalence of MAFLD and hepatic fibrosis among the study participants.

	Hepatic Fibrosis (95% CI, %)	MAFLD (95% CI, %)
total estimation	10.0 (9.3–10.7)	51.6 (50.4–52.7)
gender		
male	5.4 (4.9–5.9)	31.9 (30.6–33.2)
female	5.2 (4.6–5.7)	37.3 (36.1–38.6)
race/ethnicity		
mexican american	1.6 (1.3–1.9)	14.8 (13.7–15.8)
other hispanic	1.2 (0.9–1.5)	9.8 (8.9–10.7)
non-hispanic white	3.9 (3.5–4.4)	27.2 (26.0–28.4)
non-hispanic black	2.6 (2.2–3.0)	19.8 (18.6–20.9)
other race	1.5 (1.2–1.8)	14.0 (12.9–15.0)
PIR		
≤1	1.9 (1.6–2.2)	14.9 (13.9–16.0)
1–3	4.2 (3.7–4.7)	29.1 (27.8–30.3)
>3	3.2 (2.8–3.7)	26.0 (24.8–27.2)
BMI		
underweight	0.3 (0.2–0.4)	n.a.
normal weight	0.9 (0.7–1.1)	1.8 (1.3–2.2)
overweight	1.8 (1.5–2.2)	26.6 (25.4–27.8)
obese	7.3 (6.7–7.9)	40.5 (39.3–41.7)
smoking	2.1 (1.8–2.4)	14.6 (13.5–15.7)

Note: Data are presented as ratio with 95% CI. Abbreviations: MAFLD, metabolic dysfunction-associated fatty liver disease; PIR, ratio of family income to poverty; BMI, body mass index; n.a., not applicable.

**Table 4:** ORs with 95% CIs of different factors related to MAFLD and hepatic fibrosis.

	Model 1		Model 2		Model 3	
	OR (95% CI)	*p*	OR (95% CI)	*p*	OR (95% CI)	*p*
hepatic fibrosis						
5-mTHF	0.98 (0.98–0.99)	<0.001	0.98 (0.98–0.99)	<0.001	0.99 (0.98–0.99)	<0.001
folic acid	0.89 (0.85–0.93)	<0.001	0.91 (0.87–0.95)	<0.001	0.94 (0.89–1.00)	0.043
5-fTHF	0.37 (0.01–10.77)	0.560	0.43 (0.02–9.31)	0.587	0.09 (0.00–666.60)	0.599
THF	3.03 (2.41–3.80)	<0.001	2.40 (1.91–3.02)	<0.001	2.41 (1.65–3.52)	<0.001
5,10-mTHF	2.47 (0.54–11.32)	0.246	2.83 (0.61–13.08)	0.184	2.04 (0.21–20.21)	0.542
MeFox	1.05 (1.02–1.09)	0.002	1.04 (1.00–1.07)	0.045	0.99 (0.92–1.08)	0.880
MAFLD						
5-mTHF	0.99 (0.98–0.99)	<0.001	0.99 (0.98–0.99)	<0.001	0.99 (0.98–1.00)	0.005
folic acid	0.98 (0.96–1.00)	0.054	0.98 (0.96–1.00)	0.102	0.98 (0.95–1.01)	0.260
5-fTHF	0.03 (0.00–7.99)	0.215	0.01 (0.00–3.60)	0.127	0.00 (0.00–1.70)	0.067
THF	2.68 (2.06–3.49)	<0.001	2.50 (1.92–3.26)	<0.001	1.76 (1.27–2.43)	0.001
5,10-mTHF	0.69 (0.14–3.35)	0.644	0.71 (0.15–3.46)	0.670	2.55 (0.34–19.12)	0.362
MeFox	1.06 (1.00–1.12)	0.035	1.04 (0.99–1.10)	0.147	0.98 (0.91–1.05)	0.487

Note: Data are presented as ORs with 95% CIs. Model 1 was unadjusted, providing the crude OR. Model 2 was adjusted for age and gender. Model 3 was further adjusted for race/ethnicity, education level, family poverty–income ratio (PIR), BMI, waist circumference, and smoking status. Abbreviations: MAFLD, metabolic dysfunction-associated fatty liver disease; 5-mTHF, 5-methyltetrahydrofolate; 5-fTHF, 5-formyltetrahydrofolate; THF, tetrahydrofolate; 5,10-mTHF, 5,10-methenyltetrahydrofolate; MeFox, pyrazino-s-triazine derivative of 4-α-hydroxy-5-methyltetrahydrofolate; OR, odds ratio; CI, confidence interval.

**Table 5:** Distribution of serum folate forms.

Folate Forms (nmol/L)	Total (*n* = 7447)	Hepatic Fibrosis (*n* = 746)	*p* ^a^	MAFLD (*n* = 3840)	*p* ^b^
5-mTHF	30.90 (21.10, 46.60)	30.40 (21.00, 43.50)	0.091	29.95 (20.50, 45.70)	<0.001
folic acid	0.60 (0.46, 0.89)	0.61 (0.48, 0.90)	0.415	0.61 (0.46, 0.90)	0.123
5-fTHF	0.14 (0.14, 0.14)	0.14 (0.14, 0.14)	0.815	0.14 (0.14, 0.14)	0.438
THF	0.66 (0.47, 0.92)	0.76 (0.52, 1.04)	<0.001	0.70 (0.50, 0.96)	<0.001
5,10-mTHF	0.14 (0.14, 0.14)	0.14 (0.14, 0.14)	0.072	0.14 (0.14, 0.14)	<0.001
MeFox	1.23 (0.76, 2.10)	1.45 (0.90, 2.49)	<0.001	1.31 (0.80, 2.24)	<0.001

Note: Data are presented as the median with the first and third quartiles (25th and 75th percentiles). *p* values are calculated using the Mann–Whitney U test. *p* ^a^: The liver fibrosis group vs. the non-fibrosis group. *p* ^b^: The MAFLD group vs. the non-MAFLD group. Abbreviations: MAFLD, metabolic dysfunction-associated fatty liver disease; 5-mTHF, 5-methyltetrahydrofolate; 5-fTHF, 5-formyltetrahydrofolate; THF, tetrahydrofolate; 5,10-mTHF, 5,10-methenyltetrahydrofolate; MeFox, pyrazino-s-triazine derivative of 4-α-hydroxy-5-methyltetrahydrofolate.

## Data Availability

The data stemmed from the combination of the National Health and Nutrition Examination Survey (NHANES) 2017–2018 and 2017–2020 (March) cycles. More information is available on the website (https://wwwn.cdc.gov/nchs/nhanes/default.aspx, accessed on 30 May 2025).

## Supplementary Materials

The following supporting information can be downloaded at: https://www.mdpi.com/article/10.3390/metabo15060370/s1, Table S1: General characteristics of various subgroups; Table S2: Serum folate form levels of various subgroups.
