# Supplementary material for: The Associations of Serum Folate Forms with Metabolic Dysfunction-Associated Fatty Liver Disease and Liver Fibrosis: A Nationwide Cross-Sectional Study
Source: Metabolites. 2025 Jun 5;15(6):370. doi: 10.3390/metabo15060370 (PMC12195386; doi:10.3390/metabo15060370)
Supplement: Supplementary file 1 [file metabolites-15-00370-s001.zip › metabolites-3646438-supplementary.pdf]

Table S1. General characteristics of various subgroups.

Characteristics	Non-Hepatic Fibrosis (n = 6701)	Non-MAFLD (n = 3607)	Hepatic Fibrosis with MAFLD (n = 594)	Hepatic Fibrosis without MAFLD (n = 152)	P^c
age (year)	47±17	45±18	55±14	57±17	0.053
20–39, %	38.5	45.7	18.9	19.1	
40–59, %	32.7	28.4	39.3	28.3	
≥ 60, %	28.2	25.9	41.8	52.6	
gender, female (%)	61.4	64.6	50.5	42.1	0.064
race/ethnicity (%)					0.029
mexican american	12.9	9.6	16.3	7.9	
other hispanic	9.8	9.6	11.4	9.2	
non-hispanic white	33.6	32.7	37.0	36.2	
non-hispanic black	25.0	26.8	22.7	29.6	
other race	18.7	21.3	12.5	17.1	
education (%)					0.017
less than high school	17.5	17.4	18.5	30.3	
high school or equivalent	23.4	22.9	25.1	22.4	
college or above	33.1	32.1	38.6	31.6	
unknown	26.0	27.6	17.8	15.8	
PIR (%)					0.141
≤ 1	19.6	20.7	19.1	23.9	
1–3	42.1	40.9	44.5	48.5	
> 3	38.3	38.4	36.4	27.6	
BMI (%)					<0.001
underweight	5.3	10.5	n.a.	13.3	
normal weight	23.8	44.4	1.4	37.8	
overweight	32.4	27.4	17.1	17.5	
obese	38.5	17.8	81.5	31.5	
waist circumference (cm)	98.07±16.11	89.11±13.11	120.74±17.02	95.36±17.42	<0.001
smoking (%)					<0.001
never	54.6	47.2	66.9	36.5	
current	45.4	52.8	33.1	63.5	
drinking (%)					0.002
never	18.7	17.3	26.7	23.7	
sometimes	59.9	60.8	58.6	48.9	
often	21.4	21.9	14.7	27.5	
Hepatitis B (%)	0.8	0.7	1.2	3.9	0.048
Hepatitis C (%)	1.2	1.5	4.4	7.2	0.002
diabetes mellitus (%)	12.8	5.5	42.6	15.1	<0.001
ALT (U/L)	20.16±13.62	17.84±12.13	31.29±26.61	28.35±21.85	0.042
GGT (IU/L)	26.65±29.65	24.59±51.16	52.83±64.84	71.22±209.94	0.012
CRP (mg/L)	3.92±7.95	2.96±6.49	5.95±8.06	4.90±10.78	<0.001
CAP (dB/m)	256.49±60.07	212.76±40.04	329.29±41.73	221.99±52.95	<0.001
LSM (kPa)	4.86±1.25	5.09±4.32	14.42±10.63	16.39±16.75	0.509

Note: data are presented as mean ± standard deviation (SD) for continuous variables and proportion for categorical variables. P values are calculated using the Chi-square test for categorical variables and the Mann-Whitney U test for continuous variables. P^c: the hepatic fibrosis

with MAFLD group vs. the hepatic fibrosis without MAFLD group. Abbreviations: MAFLD, metabolic dysfunction-associated fatty liver disease; PIR, ratio of family income to poverty; BMI, body mass index; ALT, alanine aminotransferase; GGT, gamma-glutamyl transaminase; CRP, C-reactive protein; CAP, controlled attenuation parameter; LSM, liver stiffness measurement; n.a., not applicable.

Table S2. Serum folate forms levels of various subgroups.

**Folate Forms (nmol/L)**	**Non-Hepatic Fibrosis (*n* = 6701)**	**Non-MAFLD (*n* = 3607)**	**Hepatic Fibrosis with MAFLD (*n* = 594)**	**Hepatic Fibrosis without MAFLD (*n* = 152)**	P^c
5-mTHF	31.10 (21.10, 46.90)	31.80 (21.60, 47.70)	29.75 (20.68, 43.00)	31.80 (22.78, 45.50)	0.115
folic acid	0.60 (0.46, 0.89)	0.60 (0.45, 0.87)	0.61 (0.48, 0.91)	0.60 (0.47, 0.86)	0.436
5-fTHF	0.14 (0.14, 0.14)	0.14 (0.14, 0.14)	0.14 (0.14, 0.14)	0.14 (0.14, 0.14)	0.870
THF	0.66 (0.47, 0.91)	0.64 (0.45, 0.87)	0.78 (0.53, 1.04)	0.67 (0.44, 1.02)	0.051
5,10-mTHF	0.14 (0.14, 0.14)	0.14 (0.14, 0.14)	0.14 (0.14, 0.14)	0.14 (0.14, 0.14)	0.027
MeFox	1.20 (0.75, 2.06)	1.14 (0.71, 1.93)	1.47 (0.91, 2.58)	1.36 (0.86, 2.38)	0.384

Note: data are presented as the median with the first and third quartiles (25th and 75th percentiles). *P* values are calculated using the Mann-Whitney U test. P^c: the hepatic fibrosis with MAFLD group vs. the hepatic fibrosis without MAFLD group. Abbreviations: MAFLD, metabolic dysfunction-associated fatty liver disease; 5-mTHF, 5-methyltetrahydrofolate; 5-fTHF, 5-formyltetrahydrofolate; THF, tetrahydrofolate; 5,10-mTHF, 5,10-methenyltetrahydrofolate; MeFox, pyrazino-s-triazine derivative of 4-α-hydroxy-5-methyltetrahydrofolate.
